# Comparison of the Effect of Simulated Gastric Acid on the Surface Properties of Different Fluoride-Releasing Dental Restorative Materials

**DOI:** 10.3290/j.ohpd.c_2761

**Published:** 2026-07-13

**Authors:** Lizeth Romero-Cabrera, Bernardo Teutle-Coyotecatl, Abigailt Flores-Ledesma, Rosario Jiménez-Flores, María Guadalupe Sol-Gamboa, Rosalía Contreras-Bulnes, Laura Emma Rodríguez-Vilchis, Eunice de Anda-Reyes, María de los Angeles Moyaho-Bernal

**Affiliations:** a Lizeth Romero-Cabrera Dentistry Student, Faculty of Stomatology, Meritorious Autonomous University of Puebla, Mexico. Performed the experiments as partial fulfillment for undergraduate degree requirements.; b Bernardo Teutle-Coyotecatl Professor, Department of Pediatric Dentistry, Faculty of Stomatology, Meritorious Autonomous University of Puebla, Mexico. Wrote the manuscript.; c Abigailt Flores-Ledesma Professor, Dental Materials Laboratory, Faculty of Stomatology, Meritorious Autonomous University of Puebla, Mexico. Performed testing.; d Rosario Jiménez-Flores Professor, Faculty of Stomatology, Meritorious Autonomous University of Puebla, Mexico. Proofread the manuscript.; e María Guadalupe Sol-Gamboa Professor, Faculty of Stomatology, Meritorious Autonomous University of Puebla, Mexico. Proofread the manuscript.; f Rosalía Contreras-Bulnes Professor, Center for Research and Advanced Studies in Dentistry, School of Dentistry, Autonomous University of Mexico State, Mexico. Proofread the manuscript.; g Laura Emma Rodríguez-Vilchis Professor, Center for Research and Advanced Studies in Dentistry, School of Dentistry, Autonomous University of Mexico State, Mexico. Proofread the manuscript.; h Eunice de Anda-Reyesh Professor, Institute of Physics, Meritorious Autonomous University of Puebla, Mexico. Wrote the manuscript, performed testing.; i María de los Angeles Moyaho-Bernal Professor, Department of Pediatric Dentistry, Faculty of Stomatology, Meritorious Autonomous University of Puebla, Mexico. Wrote the manuscript.

**Keywords:** glass hybrid, fluoride release, simulated gastric acid, surface roughness, solubility.

## Abstract

**Purpose:**

Eating disorders such as bulimia and anorexia negatively impact on oral health due to repeated exposure to gastric acid, leading to dental erosion and deterioration of restorative materials. Resin-Modified Glass-Ionomer Cement (RMGIC), Giomers and glass hybrid may offer greater resistance in these adverse environments. This study aimed to compare the effect of simulated gastric acid on the surface properties of different fluoride-releasing composite materials.

**Materials and Methods:**

An in-vitro experimental study was conducted using disks of RMGIC (Gold Label Light-Cured Universal Restorative; G1_GLLC), Giomer (Beautifil II; G2_BFII), and glass hybrid (EQUIA Forte Fil; G3_EF), prepared according to manufacturers’ instructions. Surface roughness, fluoride release and solubility were evaluated before and after 18 h of immersion in simulated gastric acid. Additionally, surface morphology was analyzed by scanning electron microscopy (SEM). Data were analyzed with one-way ANOVA and Student’s t-test (p < 0.05).

**Results:**

G1_GLLC exhibited the highest increase in surface roughness after immersion (0.38 ± 0.25 µm), while G2_BFII demonstrated lower changes in surface roughness (0.17 ± 0.16 µm) and the lowest fluoride release (0.001 ppm) and solubility (0.93%). G3_EF showed the highest solubility (5.50%), indicating greater susceptibility to acidic environments.

**Conclusion:**

The surface roughness, fluoride release, and solubility of all tested materials were affected by simulated gastric acid. Giomer (G2_BFII) proved to be a suitable alternative in a highly acidic oral environment, as it remains stable and releases fluoride under acidic conditions compared to RMGIC (G1_GLLC) and glass hybrid (G3_EF).

In 2021, according to the Global Burden of Disease (GBD), 16 million people had eating disorders, of which almost 3.4 million were children and adolescents. Eating disorders represent a major public health concern, particularly among adolescents.^11^ Anorexia nervosa is a disorder characterized by the persistent restriction of food intake, leading to significantly low body weight relative to age, sex, and physical health. On the other hand, Bulimia nervosa is characterized by recurrent episodes of binge eating followed by inappropriate compensatory behaviors, such as self-induced vomiting, use of laxatives, fasting, or excessive exercise, to prevent weight gain. Self-esteem is also influenced by body weight and shape.^40^

These disorders cause a chronic decrease in oral cavity pH due to frequent vomiting episodes,^29^ with values ranging from 1 to 3,^30^ creating an extremely acidic environment that affects both dental tissues^27^ and the restorative materials used. This acidic environment, associated with recurrent vomiting, promotes enamel demineralization^26^ and increases the vulnerability of restorations, potentially leading to early failures. Patients suffering from anorexia and bulimia not only experience considerable dental deterioration but also face an increased risk of caries and erosions,^7^ complicating clinical management. Additionally, these problems can affect some properties of dental materials, e.g., surface roughness, which is the main cause of plaque accumulation, color change, wear, and esthetic properties of the materials.^16^ The extremely acidic environment also affects the solubility of materials, which in turn promotes biocompatibility changes, degradation, loss of marginal integrity, and dimensional changes, potentially causing a shortened functional life due to their rate of deterioration, resulting in restoration failure.^37,41^ However, in fluoride-releasing materials, the solubility is to a certain extent desirable, since this enables their bioactivity. To prevent the deterioration and degradation of dental materials exposed to frequent periods of acidity,^12^ various modified dental materials based on glass-ionomer cement (GIC) have been developed. Among them are resin-modified glass-ionomer cements (RMGICs) that can be considered bioactive restorative materials, since they release and recharge fluoride ions which in turn remineralize hard dental tissues. These are used when the operator wishes to have a controlled cure of the material.^10^

Giomer is a bioactive material containing surface pre-reacted glass-ionomer (SPRG) fillers. The use of SPRG fillers ensure fast fluoride release by ion exchange in the previously reacted hydrogel.^12^ It is considered a true hybridized restorative material of GIC and composite which has fluoride release and recharging properties.

Glass hybrid is a type of high-viscosity glass-ionomer cement (HVGIC) that exhibits less sensitivity to moisture in the early curing period, greater hardness and abrasion resistance, fluoride release, and utility as a bulk-fill material.^17^

In a highly acidic oral environment, restorative materials such as RMGICs,^34^ Giomers,^36^ and glass hybrid are considered viable options due to their ability to release fluoride, an essential element for enamel remineralization and the prevention of dental erosion. Moreover, the evaluation and selection of an appropriate material that can withstand the acidic challenges plays a key role in the durability and longevity of dental restorations. However, the impact of gastric acid on the critical properties of these materials, including roughness, solubility, and fluoride release, has not been thoroughly explored.^39,41^ Thus, to fill this knowledge gap, this study integrates surface roughness analysis, fluoride release, and solubility to evaluate the balance between bioactivity and structural stability in contemporary restorative materials. The null hypothesis was that simulated gastric acid exposure does not produce any statistically significant alterations in the surface roughness, solubility, fluoride release, and surface morphology of three fluoride-releasing dental restorative materials: RMGIC, giomer, and glass hybrid.

## MATERIALS AND METHODS

### Sample Size Calculation

This study followed the CRIS guidelines.^20^ The sample size was calculated using the G*Power software (v.3.1.9.7; Heinrich Heine University, Düsseldorf, Germany) with a significance level of 0.05, a statistical power of 95%, and an effect size of 0.80. The effect size was derived from the results of previous studies.^5,13^ The required sample size was calculated to be at least 10 samples per group.

### Materials Selection

Three fluoride-releasing dental restorative materials widely employed in pediatric dentistry for the restoration of anterior and posterior teeth were selected for this study: An RMGIC (Gold Label Light-Cured Universal Restorative, GC; Tokyo, Japan), a giomer (Beautifil II, Shofu; Kyoto, Japan) and a glass hybrid (EQUIA Forte Fil, GC). The technical details of the materials are presented in Table 1.

**Table 1:** Description of fluoride-releasing dental restorative materials used in this study

Material Group name	Manufacturer	Lot	Composition	Particle size
GC Gold-label light-cured Universal Restorative G1_GLLC	GC; Tokyo, Japan	2110231	Water, polyacrylic acid, HEMA, UDMA, and camphorquinone, fluoroaminosilicate glass (amorphus), 5% polyacrylic acid	5.9 μm
Beautifil II G2_BFII	Shofu; Kyoto, Japan	012194	Bis-GMA, triethylene glycol dimethyl ether, aluminofluoro-borosilicate glass, aluminum oxide, DL camphorquinone, others	0.8 μm (800 nm)
EQUIA Forte Fil G3_EF	GC	2012181	Aqueous polyacrylic acid, strontium fluoroaluminosilicate glass, polyacrylic acid	10 μm
HEMA: 2-hydroxyethyl methacrylate; UDMA: urethane dymethacrylate; bis-GMA: bisphenol A-glycidyl methacrylate.				

### Group Allocation

Ninety-three samples were distributed into three groups (n = 31 per group): G1_GLLC (RMGIC), G2_BFII (giomer), and G3_EF (glass hybrid). The properties by group were evaluated as follows: surface roughness test (n = 10 per group), fluoride release test (n = 10 per group), solubility test (n = 10 per group), and surface characterization by SEM (n = 1 per group). The same samples were used for measurements before and after immersion in simulated gastric acid.

### Sample Preparation

Materials were prepared at room temperature according to the manufacturer’s instructions. These materials were placed into a fabricated stainless-steel mold (diameter: 4.5 mm; thickness: 2 mm).^31^ Specimens of the Gold Label Light-Cured Universal Restorative and Beautifil II were light-cured through Mylar film using an LED curing unit (Bluephase N 100–240V, Ivoclar Vivadent; Ellwangen, Germany). The curing time was 20 s at 1200 mW/cm^2^, performed with the light-guide tip in direct contact with the upper Mylar film. The irradiance value was measured using a radiometer (Bluephase Meter II, Ivoclar Vivadent). For the Equia Forte Fil specimens, the capsule was activated just before mixing and was immediately placed in the amalgamator. The restorative material was mixed for 10 s and let cure for 2.5 min after placing it into a prefabricated stainless-steel mold. The samples were kept in distilled water for 24 h at 37°C to complete polymerization.^17^

### Immersion of Fluoride Release Materials in Simulated Gastric Acid Solution

Samples were individually immersed in 2 ml of simulated gastric acid solution (0.06 M HCl, pH 1.2) for 18 h and stored in an incubator at 37°C to simulate the 2 years of cumulative exposure to gastric acid.^13,32^

### Surface Roughness Evaluation 

A Mitutoyo profilometer (Surftest SJ-310; Tokyo, Japan) was used to perform surface roughness analysis at a speed of 0.25 mm/s and a cutoff distance of 0.08 mm 5x. The samples were fixed with double-sided tape (JANEL 400; Tlapaltitlan, Mexico) parallel to the tip of the roughness gauge. Three measurements of Ra and Rz parameters per sample were taken before and after immersion in simulated gastric acid at the same location on each sample and then averaged to obtain the final roughness values.^15^

### Fluoride Release Measurement

Measurements were conducted using a fluoride ion-selective electrode (Thermo Scientific ISE Orion electrode, Ionplus Sure-Flow Fluoride; Chelmsford, MA, USA) coupled to a benchtop analyzer (Thermo Scientific Orion 4-star Plus pH ISE, Thermo Scientific) with a detection limit of ± 0.001 ppm. The electrode was calibrated with a standard fluoride solution with concentrations of 0.1, 1, 10, and 100 ppm. Once a suitable calibration curve was observed, the readings were taken.

To quantify the fluoride concentration before immersion of fluoride release materials into simulated gastric acid, 1 ml of distilled water used to complete curing was added to 1 ml of TISAB II solution (Thermo Scientific Orion 940907 ISE). Subsequently, the fluoride ion-selective electrode was inserted into the test tube, and the fluoride concentration in parts per million (ppm) was recorded. The electrode was rinsed with deionized water before each measurement. After the immersion period (18 h at 37°C) in simulated gastric acid solution, 1 ml of that solution was combined with 1 ml of TISAB II solution to measure fluoride release.^14,23^

### Solubility Test

Specimens were weighed (m_0_) and suspended in a wire basket inside 2-ml microtubes, ensuring they remained intact. Then, incubation in simulated gastric acid was performed as described above. After the immersion period, samples were rinsed with deionized water, gently dried, and weighed again (m_1_). They were subsequently placed in a desiccator with silica gel and weighed daily until a stable weight was reached, with no variation greater than 0.001 g for three consecutive days (m_2_).^31^

The amount of solubilized material was expressed as a percentage using the formula:

S (solubility) = (m_0_ – m_2_) / m_0_

where:

m_0_ = initial weight before immersion

m_2_ = final weight after stabilization in the desiccator

### Scanning Electron Microscopy

The descriptive evaluations using scanning electron microscopy (SEM, JEOL JSM-5400LV; Tokyo, Japan) were performed on independent and representative samples from groups G1_GLLC, G2_BFII, and G3_EF. They were examined to observe surface morphology before and after immersion in simulated gastric acid. In this sense, samples were mounted on aluminum stubs using double-sided carbon adhesive tape (SPI Supplies; West Chester, MA, USA) and examined under the following conditions: low vacuum mode, chamber pressure of 10 Pa, electron accelerating voltage of 15 kV, and working distances adjusted to obtain optimal images. Surface characterization was performed at magnifications of 100X, 500X, and 1500X.^31^

### Statistical Analysis

The data obtained in this research work were analyzed with a statistical software package (SPSS.25, IBM; Armonk, NY, USA). The data distribution was evaluated with the Shapiro-Wilk test. Differences between surface roughness and fluoride release of three materials before and after the immersion in simulated gastric acid were assessed separately using one-way ANOVA and Tukey’s post-hoc test. Student’s t-test was employed to find differences in the same type of material before and after of the immersion period. Finally, one-way ANOVA and Tukey’s post-hoc test were employed to evaluate differences in solubility between the three materials after the immersion period; the margin of error was set as 0.05. The significance level was set to a p-value of < 0.05.

## RESULTS

### Surface Roughness

Average and standard deviation for roughness parameters of each material are shown in (Table 2). The group G1_GLLC showed the lower surface roughness values (Ra and Rz parameters) before immersion in simulated gastric acid contrary to G3_EF. The G1_GLLC and G2_BFII present similar surface roughness values. After immersion in simulated gastric acid all study groups present similar surface roughness in arithmetic mean roughness (Ra), however in 10-point mean roughness (Rz) parameter there are significant differences. Only G1_GLLC group show differences among surface roughness before and after immersion in simulated gastric acid.

**Table 2:** Mean and standard deviations (SD) of surface roughness test (µm), before and after simulated gastric acid solution exposure

Groups	Roughness, arithmetic mean [Ra] (µm)			10-point mean roughness [Rz] (µm)		
	Before	After	Student’s t-test	Before	After	Student’s t-test
G1_GLLC	0.06 ± 0.03^a^	0.38 ± 0.25^a^	p < 0.001*	0.32 ± 0.18^a^	3.17 ± 1.70^a^	p < 0.001*
G2_BFll	0.12 ± 0.13^a^	0.17 ± 0.16^a^	p = 0.4530	0.57 ± 0.52^a^	1.00 ± 0.84^b^	p = 0.1856
G3_EF	0.29 ± 0.17^b^	0.34 ± 0.27^a^	p = 0.6262	1.60 ± 0.86^b^	1.95 ± 1.43^a,b^	p = 0.5156
ANOVA	p < 0.001	p = 0.117		p < 0.001	p < 0.001	
*Statistically significant at p ≤ 0.05. Means with different superscript letters in columns are statistically significantly different according to the post-hoc Tukey test.						

### Fluoride Release

Before immersion in simulated gastric acid, the highest fluoride release was observed in G3_EF (11.00 ± 3.16 ppm), followed by G1_GLLC (8.60 ± 1.43 ppm), with no statistically significant differences between these groups. G2_BFII presented the lowest fluoride release (0.25 ± 0.10 ppm). After immersion in simulated gastric acid, all groups showed a decrease in fluoride release, with G1_GLLC (0.09 ± 0.02 ppm), G3_EF (0.07 ± 0.02 ppm), and G2_BFII (0.01 ± 0.00 ppm), showing statistically significant differences before and after immersion (Table 3).

**Table 3:** Fluoride release as means and SD before and after simulated gastric acid solution exposure

Fluoride release (ppm)	Before	After	Student’s t-test
G1_GLLC	8.60 ± 1.43^a^	0.09 ± 0.02^a^	p < 0.001*
G2_BFll	0.25 ± 0.10^b^	0.01 ± 0.00^b^	p < 0.001*
G3_EF	11.0 ± 3.16^a^	0.07 ± 0.02^c^	p < 0.001*
ANOVA	p < 0.001	p < 0.001	
*Statistically significant at p ≤ 0.05. Means with different superscript letters in columns are statistically significantly different according to the post-hoc Tukey test.			

### Solubility

After immersion into simulated gastric acid the G2_BFII showed the lowest solubility percentage (0.93 ± 0.39%), while G3_DFF had the highest value (5.50 ± 0.82%), indicating greater susceptibility to dissolution. G1_GLLC showed an intermediate percentage (3.28 ± 0.79%), with statistically significant differences between groups (ANOVA, p < 0.001), confirmed by Tukey’s post-hoc test (Table 4).

**Table 4:** Solubility of fluoride-releasing restorative materials

Groups (n = 10)	Solubility (%)
G1_GLLC	3.28 ± 0.79^a^
G2_BFll	0.93 ± 0.39^b^
G3_EF	5.50 ± 0.82^c^
ANOVA	p < 0.001
Means with different superscript letters in columns are statistically significantly different according to Tukey’s test.	

### SEM

**Fig 1:**
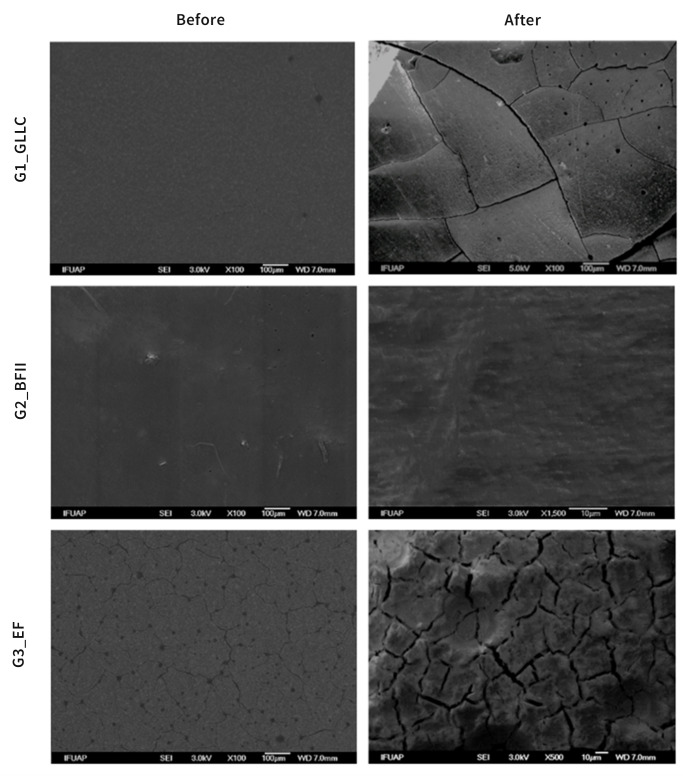
Representative SEM images of three fluoride-releasing dental restorative materials before and after simulated gastric acid. Original magnifications: 100X (a, b, c, e), 500X (f), 1500X (d); scale bar = 10 µm (d and f) or 100 µm (a, b, c, e).

The SEM micrographs of the three fluoride-releasing restorative materials taken before and after simulated gastric acid are shown in Fig 1. The SEM images of intact materials showed a smooth and homogeneous surface with inherent characteristics of each material (Figs 1a, 1c, 1e). In the SEM images taken after immersion in simulated gastric acid, group G1_GLLC exhibited marked fractures (Fig 1b), G2_BFII showed slight fissures, maintaining better surface integrity (Fig 1d). G3_EF showed greater deterioration with deep fractures, suggesting higher susceptibility to acid attack (Fig 1d).

## DISCUSSION

Fluoride-releasing dental restorative materials such as glass hybrid, RMGICs, and Giomers have been widely used in pediatric dentistry due to their adhesive and bioactive properties, as well as fluoride-releasing ability;^18,27^ however, its behavior under highly acidic conditions, such as those present in patients with eating disorders (bulimia and anorexia), has rarely been explored.^25^ These patients are repeatedly exposed to gastric acid due to vomiting, which increases the risk of dental erosion and deterioration of restorative materials. In this research, three key physical properties, such as surface roughness, fluoride release, and solubility were evaluated before and after exposure to simulated gastric acid. These parameters are essential to determine the resistance of the material in adverse environments, as well as their therapeutic potential and clinical durability. Considering that a patient suffering from bulimia has at least 3 vomit episodes daily, and that the contact time between vomit and restoration material is under 1 min, it can be estimated that the immersion time used in this study mimics the effects of 2 years of gastric juice exposure.^13^ This provides new insights into the performance of fluoride-releasing materials under extreme intraoral conditions.

The surface roughness analysis was carried out using contact profilometry because it is a reliable, simple method that has been employed in some studies,^1,5,12^ in addition to being regulated by international standards (JIS B 0651:2001).^15^ Optical profilometry was not used in this study because it still presents several recognized problems, most notably the effect of color and transparency on the results of hard tissues and dental materials.^9^

According to the contact profilometry results, before the immersion of the materials, G3_EF was the roughest material followed by G2_BFII and finally G1_GLLC. This result could be related to the particle size of each material, since recent literature indicates that the surface roughness of dental materials is proportional to their particle size.^29^ Equia Forte has the largest particles (10 µm), followed by GC Gold Label (5.9 µm) and finally Beautifil (0.8 µm).^6,12^

After the immersion period, only G1_GLLC presented a great increase in its surface roughness in a range of 633% to 990% in relation to the evaluated roughness parameter. This behavior coincides with that reported by Abakli et al,^1^ who showed that RMGICs present an increase in roughness after contact with acidic media. This surface alteration could be explained by the susceptibility of the glassy matrix and the particle size in the ionomers,^35^ which favor surface degradation at low pH.^19,38^ The degradation of GIC is a complex process of absorption, disintegration, and outward transportation of ions. In this sense the H^+^ ion of simulated gastric acid could be spread from the solution to the material and is replaced by Ca^+2^ or Al^+3^ ion in the matrix. As the amount of metal cations in the matrix decreases, dissolution increases around glass particles; this phenomenon contributes to the increase in surface roughness due to the pits formed by the dislocation of glass particles and the ledges formed by undissolved glass particles.^6^

As can be seen, the roughness of Giomer did not statistically significantly increase, which could be due to greater stability attributed to the presence of a resin matrix reinforced with pre-reacted fillers (S-PRG) that contribute to a more erosion-resistant microstructure.^41^ Ünal et al^39^ found that the Giomer showed less roughness after being exposed to gastric acid, which is consistent with the results obtained in this research.

Finally, G3_EF also did not show statistically significant changes in surface roughness. It retained its initial values, contrary to what was reported by Willers et al,^41^ who found a statistically significant increase in surface roughness and surface loss after exposure to hydrochloric acid; this was probably due to the longer immersion time, as they incubated their samples for 30 h. In the present study, the samples were not coated with EQUIA Forte Coat because fluoride release was also analyzed, and this property can be affected by the resin matrix of the coating. The literature mentions that applying the coating inhibits fluoride release.^2^

Rough surfaces foster bacterial plaque accumulation and increase the likelihood of abrasion, which reduces the gloss of the restoration and increases the risk of discoloration. The average roughness values reached after immersion in simulated gastric acid are above the threshold (0.2 µm) considered favorable for the accumulation of microorganisms,^3^ yet they do not exceed the values (0.5 µm) perceived as uncomfortable by oral tissues.^33^ Furthermore, the roughness values obtained (0.06 to 0.29 µm) are within the range considered low (0.7 to 3.4 µm).^43^

Fluoride release is an essential parameter due to its anticariogenic properties.^22,27^ In this sutdy, before immersion the materials presented different amounts of fluoride release. G1_GLLC released the highest quantity of fluoride, followed by G3_EF and G2_BFII. These results are in line with those of Manjunath et al,^23^ who observed that glass-ionomer-based materials (RMGIC and glass hybrid) exhibited a greater amount of fluoride release compared to Giomer when incubated for 24 h in deionized water; such findings are similar to those found in the materials evaluated in this research, which were cured in deionized water (24 h) before being immersed in simulated gastric acid solution. Additionally, the same trend in fluoride release by similar type of materials (RMGICs, GIC, and Giomer) were seen by Islam et al in deionized water.^14^

After immersion, the same trend was observed in the release of fluoride. All materials presented differences in fluoride release before and after immersion. G2_BFII released the least amount of fluoride with the highest percentage (4%) compared to G1_GLLC (1.04%) and G3_EF (0.6%). G2_BFII exhibited a more sustained and stable release over time, which could be advantageous in clinical contexts with recurrent acid exposure.

Currently, the amount of fluoride released from dental materials remains a controversial topic, as they release less fluoride compared to toothpaste. Although GICs release a significant amount of this ion, it is not enough to precipitate as calcium fluoride because it must reach a concentration above 8 ppm.^28^ Furthermore, fluoride release from GIC-based materials does not maintain elevated levels; it decreases over time.

Regarding solubility, it can be observed that RMGIC and glass hybrid presented the highest dissolution percentages. This trend agrees with that reported by Bueno et al,^4^ who observed that materials such as GC Gold Label 2 and Equia Forte eroded under acidic conditions, with Equia Forte being more affected than GC Gold Label 2.

G2_BFII presented the lowest values among all the materials evaluated, which indicates greater resistance to dissolution in acidic media. This result could be related to its hybrid structure, composed of S-PRG particles coated with a treated resin matrix^24,41^ that reduces the penetration of H^+^ ions and prevents material degradation.^37^ In contrast, RMGICs showed greater solubility, which may weaken the restoration, facilitate marginal leakage, and increase the risk of clinical failure, as has been noted in previous studies.^8,37^

Looking at the SEM images taken before the immersion into simulated gastric acid, the G3_EF group presented a non-homogeneous surface with fine cracks, in contrast to the G1_GLLC and G2_BFII groups, which presented smooth and homogeneous surfaces. Despite the application of the protective coat, Karakas et al^17^ also observed fine cracks on the surface of Equia in samples not treated under acidic media. Therefore, the fine cracks observed in the morphological analysis of the samples were not due to desiccation, as the samples were analyzed immediately after removal from deionized water. Furthermore, the samples were prepared independently and exclusively for this purpose.

After exposure to acid, the surfaces showed the presence of microfissures and cracks in all materials tested. Morphological changes were more evident in G1_GLLC (RMGIC). These findings are in line with previous studies,^1,6,12^ which found that surface roughness increased and deep cracks and pits were formed by the dissolution effect of simulated gastric acid on the glass-ionomer matrix. These structural alterations compromise the integrity of the material, reflecting its lower resistance in highly acidic environments. On the other hand, G2_BFII showed less deterioration, which reinforces its possible use in patients with bulimia or gastric reflux.

G3_EF displayed cracks and gaps, as similarly noted by Abakli et al,^1^ Willers et al,^41^ and Karakaş et al.^17^ This material had greater susceptibility, limiting its applicability in the clinical context discussed here.

The null hypothesis was partially accepted because the immersion of samples of three fluoride-releasing dental restorative materials – RMGIC, Giomer, and glass hybrid – in simulated gastric acid produced statistically significant alterations in the surface roughness, solubility, fluoride release, and surface morphology.

Among the limitations of the study is the in-vitro design, which does not fully replicate the complex and dynamic oral environment, e.g., pH fluctuations, salivary buffering capacity, enzymatic activity, mechanical abrasion by mastication or toothbrushing, and intraoral temperature variations. However, one of this study’s strengths is the inclusion of materials that are clinically relevant in pediatric dentistry.^16^ The combined evaluation of roughness, fluoride release, and solubility offer a comprehensive view of the behavior of these materials in acidic environments.

Taken together, the findings suggest that Giomer exhibits superior performance over RMGICs and glass hybrid in terms of surface stability, low solubility, and controlled fluoride release. These properties position it as a suitable option for restorations in patients with frequent exposure to gastric acid, where material integrity and protection against caries are crucial factors for long-term clinical success. In terms of clinical decision making, this should be taken into account when confronted with a very acidic oral environment. However, more clinical trials are needed.

## CONCLUSION

The simulated gastric acid had an impact on the solubility, fluoride release, and surface roughness of all examined materials. However, compared to both types of glass-ionomer cements (RMGIC and glass hybrid), the Giomers tested showed greater resistance to the simulated gastric acid. Out of the three materials, Giomers exhibited the least amount of roughness and changes in fluoride release, whereas RMGIC was the most impacted.
